# 肺癌药物临床试验中研究护士的角色职能与作用

**DOI:** 10.3779/j.issn.1009-3419.2022.102.30

**Published:** 2022-07-20

**Authors:** 富杰 郝, 琴 祝, 素娥 王, 亚 刘, 琳 姜, 瑞丽 潘

**Affiliations:** 1 100144 北京，中国医学科学院，北京协和医学院护理学院 School of Nursing, Chinese Academy of Medical Sciences and Peking Union Medical College, Beijing 100144, China; 2 100010 北京，中国医学科学院，北京协和医学院，北京协和医院呼吸与危重症医学科 Department of Respiratory and Critical Care Medicine, Peking Union Medical College Hospital, Chinese Academy of Medical Sciences and Peking Union Medical College, Beijing 100010, China

**Keywords:** 药物临床试验, 肺肿瘤, 研究护士, 角色职能, Drug clinical trials, Lung neoplasms, Clinical research nurse, Role and function

## Abstract

抗肿瘤药物临床试验既是新药研发的必经之路，也是最前沿的恶性肿瘤治疗方法，给患者带来了更多生存机会。肺癌药物临床研究数目大，研究药物品种繁多，研究进展快，临床转化效率高，这些试验的成功开展离不开研究团队的共同努力，其中研究护士也发挥了不容小觑的作用。结合临床研究护士的工作内容，本文详细介绍了开展肺癌药物临床试验过程中研究护士的岗位管理、角色职能、核心能力及职业发展前景，以期为更多医疗机构开展相关工作提供参考，促进临床研究护士向规范化专业化深入发展。

## Abstract (en)

Clinical trials of anti-tumor drugs is not only the important way to develop new drugs, but also the most advanced treatment methods for malignant tumors, bringing survival benefits to patients. There are a large number of new anti-tumor drug clinical trials for lung cancer patients, covering a wide variety of anti-tumor drugs, and with rapid progress and high efficiency of clinical transformation. These trials could not be carried out successfully without the joint efforts of the research team, in which the research nurses also played a role that should not be underestimated. Combined with the work content of clinical research nurses, this paper introduced the post management, role function, core competence and career development prospect of clinical research nurses in the process of carrying out clinical trial of lung cancer drugs in detail. In order to provide reference for more medical institutions to carry out related work, and promote the further development of clinical research nurses to standardization and specialization.

肿瘤已成为威胁我国人民健康的重大公共卫生问题，中国医学科学院国家癌症中心研究指出，预计2022年我国将有482万肿瘤新发病例和321万肿瘤死亡病例，其中肺癌的新发病例（87.1万例）和死亡病例（76.7万例）位居我国癌症之首^[1]^。药物临床试验既是新药研发的必经之路，也是恶性肿瘤最前沿的治疗方法。2020年我国登记注册的肿瘤药物临床试验共722项，比2019年增加了52.3%，其中肺癌药物临床试验位居第二，占比高达15.0%^[2]^。高质量的药物临床试验离不开试验机构、研究人员、申办方的共同努力，需要研究者、统计学家、临床监查员、临床试验协调员、临床研究护士（clinical research nurse, CRN）等通力协作。

随着肺癌药物临床试验的蓬勃发展，临床试验质量要求也进一步提高，对临床研究护士的需求也越来越大，他们的参与和协调对于临床试验的成功开展必不可少。美国国立卫生研究所将临床研究护士定义为致力于药物临床试验并承担受试者护理与协调工作的专科护士^[3]^，明确了CRN的实践领域涵盖在临床试验执行和协调中的一切护理活动。我国CRN的发展起步较晚，和普通临床护士相比，CRN的基本知识、技能和素养更强调统筹协调、科研意识和理念。正规的教育、明确的角色描述、清晰的职业发展路径对CRN在临床试验中发挥更专业的作用，以及对其职业发展和职业认可都会产生重要影响^[4]^。因此，我们需要不断明确临床研究护士的角色与职能，对研究护士进行规范化的遴选、培养、有针对性地提升其核心能力。在CRN帮助研究者发现新的研究问题以及探索新方法的同时，逐步开展临床循证护理实践，这不仅能更好地促进肺癌医疗领域的健康发展，提高护理质量并促进临床试验的顺利开展，还扩宽了护理服务的范畴，促进并推动这一新兴专科护士角色进一步向专业化规范化纵深发展。

## 1 我国肺癌药物临床试验开展概况

随着肿瘤学科的发展，抗肿瘤新药的不断出现，促使肿瘤药物临床试验进入快速发展阶段。在国际形势、国内政策、临床需求共同驱动下，自2009年肿瘤药物临床试验广泛开展，2016年后急剧增多，其中，肺癌是2009年-2018年我国注册肿瘤药物临床试验中最常见的癌症类型^[5]^。据国家癌症中心统计，2005年-2020年我国共注册肺癌药物临床试验1, 595项，2013年-2020年试验数量越来越多，年增长率达26.5%，以靶向药和免疫药为代表的大量创新性药物进入试验及临床，主要负责单位从2005年的1个增加到2020年的110个^[6]^。

## 2 肺癌药物临床试验中研究护士的角色职能与作用

### 2.1 岗位管理

出于保护医院信息资源和受试者隐私、保持研究队伍的稳定性、提高临床试验质量的考虑，许多医院已经开始逐步培养专职CRN，CRN队伍的扩大也促使着管理层思考如何提升其整体素质，做好岗位管理。对核心能力的评估是岗位管理的重要内容。屈欢等^[7]^在2015年运用扎根理论探究了肿瘤专业CRN职责、岗位胜任力评价指标，将CRN的职责分为两个基本要素（临床实践和研究管理）和一个发展要素（贡献科学），CRN岗位胜任力的评价指标为药物临床试验专业知识、临床试验管理能力、协助制定与执行能力、肿瘤专业护理技能。

#### 2.1.1 培养遴选、严格准入

为确保高效完成试验相关工作，有必要设置CRN岗位并确立具体标准进行人员选拔：①具备本科及以上学历、护师及以上专业技术职称；②从事肺癌专科护理工作≥3年；③顺利通过科室及医院组织的专业知识、实践技能、沟通能力、人文关怀、处理突发事件的培训及考核；④为增进团队科研意识，近5年有专业论文发表、参与书籍编写、科研课题申报、参加境内外培训、获得专科护士证书者优先考虑。

#### 2.1.2 完善培训、评价考核

国内学者李燕^[8]^构建的CRN核心胜任力评价指标包括基本知识、技能和素养、临床实践、研究管理、研究的协调管理和持续管理、受试者保护、科学贡献，为充分发挥CRN的核心胜任力，应完善对CRN的培训，严格准入。CRN首先要完成国家食品药品监督管理总局认可的药物临床试验管理规范培训（good clinical practice, GCP）全部课程，取得GCP证书；其次，需要完成医院药理中心组织的专项培训，通过考核获得参与院内临床药理试验的资质证书；此外，项目正式开展前，CRN要参加试验项目启动会，接受申办方进行的相关培训，熟悉试验方案、明确自身定位职责；最后，研究团队组长负责考核并确认CRN对研究方案、试验过程、药物作用、不良反应处理、标本采集及保存等掌握情况。上述一系列培训过程可以概括为“国家-医院-申办方-科室”四位一体，有助于CRN充分掌握药物临床试验及执行试验的知识和技能并做好文件准备等工作。

#### 2.1.3 明确职责、规范流程

根据国家药品监督管理局与国家卫生健康委员会2020年组织修订的《药物临床试验质量管理规范》^[9]^，设定CRN岗位后，重新制定其工作职责、内容以及具体工作流程，保证药理试验进行中护理工作的规范化及同质性。工作内容的确立以顺利开展试验及关注受试者疗效为出发点，确保过程符合药物临床试验质量管理规范要求，符合伦理性和科学性的核心宗旨，主要涵盖临床实践、研究管理、科学贡献三个维度。其具体职责包括：①参加项目方案、操作规程、急救技能等培训；②参与研究方案的评估，为开展试验提供必要的客观条件及物资准备、质量保障；③为受试者提供连续的临床护理、保护受试者的安全、维护受试者的知情同意、确保方案的完整实施、参与后续随访；④协助评估疗效、毒性反应、追踪记录不良事件并处理不良事件；⑤参与试验文档管理，记录填写试验资料，保证数据完整、资料安全；⑥根据项目规程进行试验药品、仪器设备、基础耗材等的检查清点，发放回收；⑦按要求实施各项操作，正确采集、处理、保管临床标本；⑧进行各方联络与协调管理^[10-12]^。

#### 2.1.4 激励保障、促进成长

CRN工作具有一定的特殊性，需要兼顾临床护理工作以及肺癌药物临床试验的各个流程和环节，所以对整体护理服务提出了较高的要求^[13]^。除了基本知识、技能和素养方面，更加注重其科研意识及科研理念的培养，强调统筹协调的能力。为避免工作负荷重、能力要求高对CRN造成压力，研究团队应设有激励保障机制。首先，CRN可以优先享有学习肺癌药物临床试验专题课程、肺癌专科知识培训、选送专科护士的权益；其次，根据CRN的职称、学历，分层级设置绩效奖励；第三，综合考虑CRN负责的试验项目数量、工作质量以及工作态度，作为评优和晋升的参考指标之一。设置激励制度，调动CRN工作积极性，促进其不断成长，达到激励员工和提升工作效益的作用。

### 2.2 受试者管理

受试者管理包括开展试验前的准备工作、准确用药、观察并随访受试者用药后的反应及不良事件。CRN首先辅助研究团队完成受试者招募注册、筛选入组等准备工作。确定符合试验入组的受试者后协调安排受试者完成相关诊查及支持治疗项目。

关注药物的安全性和有效性，监测、记录和上报用药后不良事件，保障患者安全是临床试验的关键组成部分^[14]^。近些年，以免疫检查点抑制剂为代表的免疫疗法成为肺癌突破性疗法之一，极大地改变了肺癌患者的治疗前景^[6]^，临床中涉及免疫治疗的试验项目也越来越多，与此同时，免疫治疗不良反应（immune-related adverse events, irAEs）也成为研究热点。CRN根据方案要求精准把握受试者用药时间及剂量、做好受试者和家属健康教育、严密监测、发现并及时上报和记录不良反应，采取相应处理措施。irAEs不同于常规化疗，会累及全身各个脏器系统，并对患者后续治疗及生活质量造成不良影响，如皮疹、瘙痒、腹泻等^[15, 16]^，严重时甚至危及生命，如免疫相关性心肌炎^[17]^。因此，CRN要在用药的整个过程中及用药后向受试者详细讲解自我观察的重点内容，使受试者对irAEs有基本的认识，以便识别不良反应并及时上报医护人员，预防irAEs的发生发展。如遇严重不良反应，按照急救预案抢救处理，全程对受试者进行照护及行为指导，不仅协调了试验研究和临床活动，并且更好地完成了与临床护士工作的衔接^[8]^。

在项目实施过程中，根据不同的临床试验类型进行分工，如在双盲双模拟药物临床试验中，受试者、研究者、数据管理员均不知治疗分配情况，盲态保持需贯彻整个试验过程，因此需设立非盲态和盲态CRN。非盲态CRN主要负责药品管理，包括药品的接收、储存、发放和配置，配置药物时应在单独的治疗室，期间不允许他人进入，以防破盲；需保证配置好的药物在外形、包装、标签和其他特征均一致方可交接给盲态CRN。由盲态CRN管理受试者，进行药物使用、病情观察、标本采集、数据收集记录及随访沟通。非盲态CRN不得接触受试者，还应杜绝非盲态CRN和盲态CRN之间的互动^[10, 18]^，以保证双盲原则始终贯穿试验过程，确保伦理合理性、科学性和试验数据的可信度。

此外，CRN应与受试者建立良好的信任关系，对于定期访视的受试者，提前与之沟通，确定访视时间，保证访视时段专人护理并管理受试者。因肺癌药物治疗具有周期性，首次发药时间决定后续随访时间，故掌握首次发药时间，有助于开展随访工作，CRN的参与使得随访安排更有条理性^[19]^；CRN还应具有应对非计划访视的能力，即受试者出现突发状况时，可以及时和CRN取得联系，保证在院及非在院期间用药安全^[20]^。

### 2.3 药品、物资、样本管理

#### 2.3.1 药品管理

肺癌治疗方案的多样性使得药物临床试验也日益复杂和精细化，通过对比借鉴国外试验用药管理相关经验^[21, 22]^，结合相应试验要求并遵循GCP及相关法规的基础上，建立并完善试验药品管理要求。CRN一般需同时担任临床试验的药品管理员，项目启动前CRN应与申办方沟通，确认使用药品种类、数量、存储条件，配备试验专用恒温柜及冰箱以满足不同药物的存储要求，另需配备隔离柜及便捷转运箱，保证特殊情况下转运及隔离药品使用。所有药物数量、有效期定期清点登记，每日监测并记录药品存储温度，如出现超温现象，首先要紧急隔离药品，随后立即联系申办方，等待下一步研判处理。CRN在受试者用药前与申办方沟通，进行随机药物编号，遵医嘱确认发药种类、剂量、熟知药物配制、输注要求、用药时严格双人核对。配置后剩余药品应遵循不同项目药品使用规程，对药盒、药瓶及标签按照药品处理规范做好回收和登记工作。

#### 2.3.2 物资管理

药物临床试验受到申办方大力支持，研究团队应根据实际需求审批经费，购置文件资料、抢救设备、生命体征测量设备、离心机、冰箱等。所有物资设置专区存放，按文件资料类、仪器设备类、试剂类、耗材类等分类统筹管理。定期进行文件整理归档，仪器设备清点登记，如冰箱、温度计等需按时进行校准备案，消耗类物资及时联系申办方请领配送。

#### 2.3.3 样本管理

生物样本管理是肺癌药物临床试验中研究者最为重视的管理工作之一，由于不同种类药物以及试验要求的复杂性，样本管理也变得较为复杂^[14, 23]^。遵照《药物临床试验生物样本分析实验室管理指南》^[24]^各项要求，研究团队应重视生物样本的离心过程，严格把控温度、转速、时间等条件，时刻关注样本贮存以及转运温度，涉及生物样本寄送时，提前与第三方实验室进行联系，确保转运过程顺利进行。

### 2.4 配合质控和质检

各项肺癌药物临床试验在开展的不同阶段均需接受国家食品药品监督管理局的督导检查，监查研究人员是否严格按照研究方案及标准操作流程开展研究。有调查^[25]^显示，药物临床试验的风险主要存在于药品及生物样本管理、试验方案设计等方面。因此，试验申办方、研究者、临床试验机构管理组织应彼此联动，进行动态化、系统化的定期监控自查，以预防及减少风险事件的发生。

## 3 CRN在肺癌药物临床试验中的优势

目前很多医疗机构的CRN从优秀临床护士中进行选拔，这确保了CRN更加熟悉临床环境及药物临床试验内部要求，熟知试验的各个环节，包含起始阶段、实施阶段及结束阶段的每一步流程，便于实施精细化管理^[26]^。在科室设置CRN，不仅明确了其工作性质、内容、责任，也增强了CRN的岗位归属感^[27, 28]^，同时服从医院、科室和研究团队的统一领导和安排，分担了更多研究者的基础工作，成为研究者的有力助手。

CRN具有扎实的疾病治疗、护理的专业知识和技能，同时具备教育、协调、管理等能力^[28]^。尤其在肺癌新药的临床试验中，负责对患者评估教育、症状观察、不良反应监测随访等工作，体现出CRN更善于提供本专业精准护理的优势。作为与受试者接触最多的群体，职能更为全面，遵循以患者为中心的理念，充分考虑受试者的社会角色、家庭角色等问题，在保证受试者依从性的同时有效提升了护患配合度，有助于试验顺利完成。

当前，肿瘤护理学科和国际临床研究护士协会逐渐明确了临床研究护理的实践范围，CRN在肿瘤学中变得越来越重要，这也使CRN的工作变得有证可循，并逐渐成为临床试验不可或缺的重要力量。

## 4 小结

临床研究护理是一个新的护理实践领域，旨在为受试者提供护理服务并确保临床研究方案的准确实施^[19]^。研究护士作为研究团队中的重要成员，在临床实践、研究管理、科学贡献等方面为保障肺癌药物临床试验的高效开展发挥着不可或缺的作用。

但是作为新兴角色，目前CRN在临床试验的参与以及病房建设和管理方面尚缺乏丰富的经验，我国CRN的实践内容更多是从事药物发放、标本采集等临床护理工作，缺乏足够的机会发展其核心胜任力，急需加快护理人才队伍建设以满足药物临床试验工作的更高需求。近年来临床试验分支逐渐细化，不同临床试验类型、不同人群、不同试验阶段各有其侧重点，护理管理人员也应结合国内药物临床试验和研究护士的发展背景和特点，借鉴国外成功经验，结合我国国情，制定并完善适合我国临床研究护士的规范化及标准化的培训课程、考核评价标准、职业发展路径等，增强护士的岗位归属感和职业认同感，使临床研究护士成为一个独特的护理专科实践领域，为临床护士的专业发展提供新的思路，并推动其不断向纵深发展。
